# An Autonomous Mobile Combination Disinfection System

**DOI:** 10.3390/s24010053

**Published:** 2023-12-21

**Authors:** Zifan Yao, Na Ma, Youdong Chen

**Affiliations:** 1 School of Mechanical Engineering and Automation, Beihang University, Beijing 100191, China; yaozf127@gmail.com; 2 Beijing Advanced Innovation Center for Biomedical Engineering, School of Biological Science and Medical Engineering, Beihang University, Beijing 100191, China; by2210221@buaa.edu.cn; 3 Key Laboratory of Biomedical Engineering and Translational Medicine, Ministry of Industry and Information Technology, Research Center for Biomedical Engineering, Medical Innovation & Research Division, Chinese PLA General Hospital, Beijing 100853, China

**Keywords:** autonomous mobile robot, UV, aerosol, combination disinfection, disinfection efficacy

## Abstract

To address the common drawbacks of current disinfection robots, which include the potential for secondary environmental pollution, disinfection dead corners, and low efficiency, in this paper, an autonomous mobile combination disinfection system is proposed. The system utilizes ultraviolet (UV) radiation and a low-concentration hydrogen peroxide aerosol to kill pathogens. It comprises three parts: a human–computer interface, a mobile robot, and disinfection equipment. A disinfection process model with continuous and fixed-point modes was established, and the effective disinfection range, speed, and duration were quantitatively calculated. The developed prototype was tested on-site by a professional third-party testing agency. The experimental results demonstrated that the combination disinfection robot achieved a 92.95% disinfection rate of natural airborne bacteria in a room measuring 22 square meters with a height of 2.8 m in just 30 min. The disinfection efficiency is at least 25% higher compared to standalone UV lamp disinfection and also exhibits a noticeable improvement over standalone hydrogen peroxide aerosol disinfection. The system enables the environmentally friendly, rapid, efficient, and all-encompassing disinfection of natural airborne bacteria. Finally, various disinfection solutions and recommendations for different application scenarios and requirements are provided.

## 1. Introduction

The ongoing emergence of new infectious diseases had a profound impact on human life, health, and social and economic development. Since the outbreak of the coronavirus disease 2019 (COVID-19) pandemic, the global economy has been severely affected, and many small- and medium-sized enterprises have been pushed to the brink of collapse. According to the World Health Organization (WHO), as of 23 December 2022, a total of 651,918,402 confirmed cases of COVID-19 and 6,656,601 deaths have been reported globally [1].

With the advancement of technology, robots and artificial intelligence have a crucial role in epidemic prevention and control. Robots can replace humans to perform highly repetitive and hazardous tasks with high quality, precision, and efficiency by utilizing intelligent algorithms. The use of robots for environmental disinfection can effectively interrupt the transmission of viruses. Disinfection can generally be categorized into two types: UV disinfection and chemical disinfection [2]. UV disinfection has been adopted since the 19th century [3]. It is a convenient, efficient, and environmentally friendly disinfection technology with minimal side effects on the objects. The wavelength (λ) of UV light ranges from 100 to 400 nm, and UV light in the UV-C band (200 nm < λ < 280 nm) can destroy the deoxyribonucleic acid (DNA) and ribonucleic acid (RNA) of microorganisms to effectively kill bacteria [4]. However, the disinfection efficacy will sharply drop when a certain distance is exceeded due to the substantial weakening of UV radiation intensity [5]. In addition, UV rays cannot pass through non-transparent surfaces, which can easily cause uneven and incomplete disinfection. Chemical disinfection is performed by spraying or atomizing a disinfectant with strong oxidizing properties. Commonly spraying disinfectants can lead to the achievement of comprehensive disinfection, but there are many disadvantages, such as waste, wetting, or even corrosion, as well as secondary pollution to the environment. Aerosol disinfection is a new type of disinfection in which the disinfectant is atomized into liquid particles with a diameter of less than 50 μm and suspended in the air before spreading in a Brownian motion for disinfection purposes [6]. Due to their good diffusibility and permeability, aerosols have many advantages, such as their high efficiency, economy, reliability, non-wetting nature, and capacity for comprehensive disinfection. Common aerosol disinfectants include hydrogen peroxide (H_2_O_2_), peracetic acid (CH_3_COOOH), chlorine dioxide (ClO_2_), hypochlorous acid (HOCl), and others. H_2_O_2_ is a safe, affordable, and easily accessible disinfectant. Moreover, it is environmentally friendly, leaving little residue after disinfection and decomposing into water vapor and oxygen. The disinfection efficacy of H_2_O_2_ aerosols is obviously better than that of common watering can spray, as ascertained through comparative experiments [7], and comparative experiments have also shown that the disinfection efficacy of H_2_O_2_ aerosols is significantly better than that of CH_3_COOOH aerosols [8].

Most of the current disinfection robots use a single disinfection method. Ma et al. [9] designed an autonomous mobile disinfection robot that can only effectively disinfect a specific small area, not the entire environment. Ruan and Chio et al. [10,11] developed an autonomous mobile disinfection robot based on H_2_O_2_ steam, but the concentration of H_2_O_2_ used was quite high (6%), which could easily cause secondary pollution to the environment and harm to human health after disinfection. The intelligent disinfection robot designed by Zhao et al. [2] achieves disinfection by spraying disinfectant. Although it can quickly and efficiently kill bacteria, there are many drawbacks, such as waste, wetting, and secondary pollution. Hong et al. [12] determined parameters such as the moving speed of the UV lamp autonomous mobile disinfection robot and the distance between the sample and the robot through comparative experiments.

To address the aforementioned drawbacks, this article proposes a combination disinfection system that primarily uses UV radiation for disinfection, supplemented by a low-concentration hydrogen peroxide aerosol. A model of the UV radiation disinfection process was established, and a quantitative analysis was conducted to determine the optimal disinfection speed and duration of the disinfection robot. Lastly, a field test was conducted to demonstrate the effectiveness of the combination disinfection system.

## 2. System Design

### 2.1. Hardware Design

**Figure 1:**
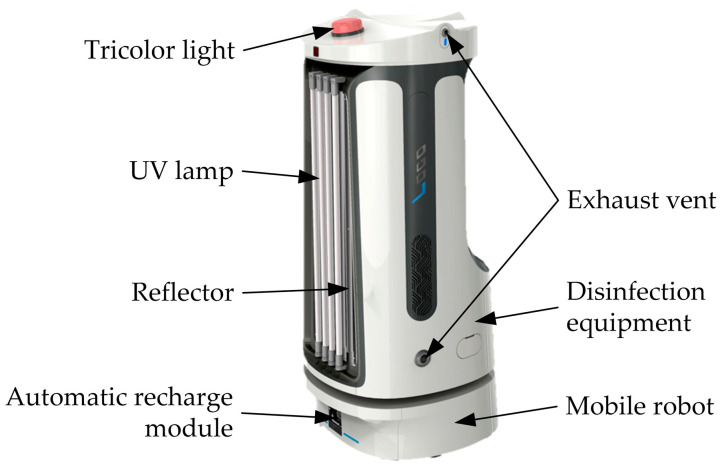
Autonomous mobile combination disinfection robot.

The autonomous mobile combination disinfection robot consists of a mobile robot and disinfection equipment, as shown in Figure 1. The mobile robot is equipped with an industrial control computer, control boards, a laser radar, an Inertial Measurement Unit (IMU), wheel encoders, and an automatic recharging module. These components collectively enable functionalities such as map construction, localization, and navigation. The mobile robot employs a dual-wheel differential drive system and, with the assistance of three caster wheels and a clever suspension structure design, is capable of smoothly navigating on flat surfaces while carrying disinfection equipment of a certain weight. The disinfection equipment includes UV lamps, a ballast, a reflector, a liquid medicine tank, ultrasonic atomizers, and exhaust vents. UV lamps are arranged in parallel at the forefront of the robot, and a reflector is added to the rear to enhance the utilization efficiency of UV radiation. To mitigate the risk of secondary pollution to the environment and potential harm to human health from disinfectants, a disinfection solution with a concentration of only 1% H_2_O_2_ is employed, which can be applied directly to human skin and mucous membranes with minimal harm, resulting in a reduction in residual disinfectant and ensuring no secondary contamination. Following the completion of the disinfection process, individuals can promptly return to the disinfected area. The atomizers are symmetrically arranged in the liquid medicine tank. The H_2_O_2_ aerosol produced by the ultrasonic atomizer is blown out of the liquid medicine tank by the exhaust vents before entering the air through the exhaust pipe. When the disinfection robot is working, the tricolor light on the top can display the working status of the robot in real time so that the operator can respond to abnormal conditions in time.

### 2.2. Software Design

**Figure 2:**
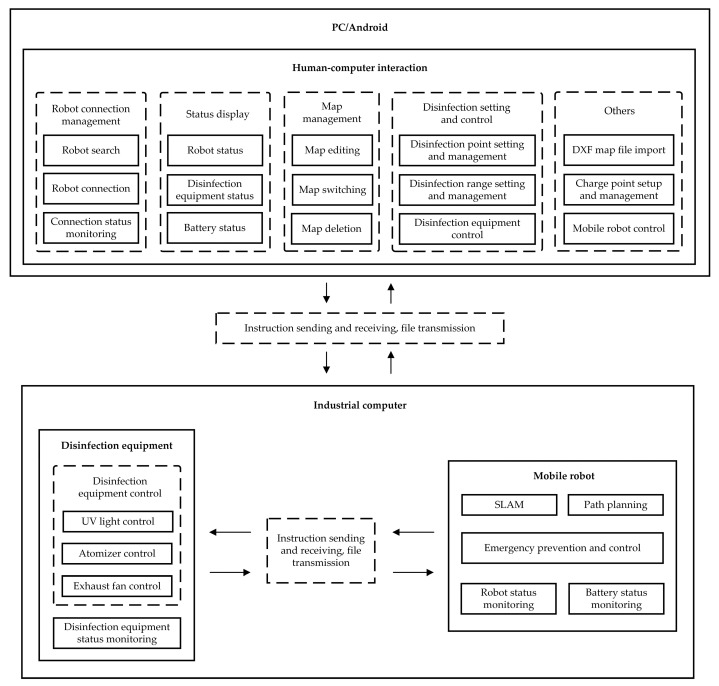
Schematic diagram of the autonomous mobile disinfection robot system.

**Figure 3:**
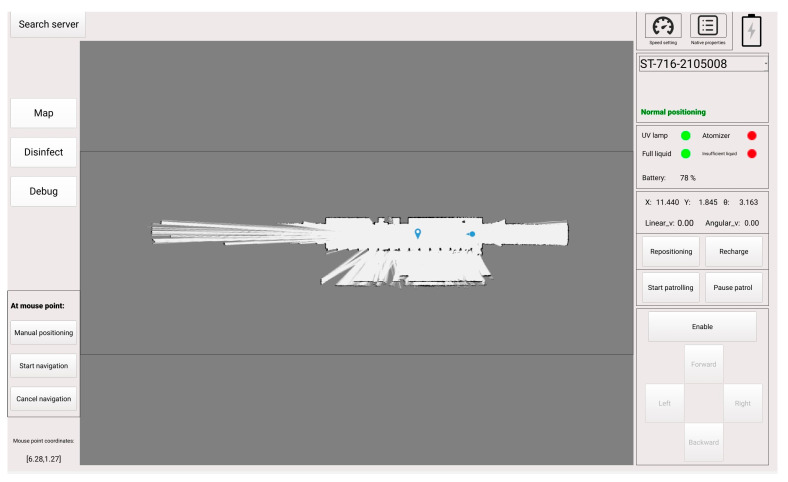
The main human–computer interface.

The autonomous mobile combination disinfection system is composed of three parts: a human–computer interface, a mobile robot, and disinfection equipment. The overall system is shown in Figure 2. To enhance the monitoring of the disinfection robot, the human–computer interface was designed and developed for both PC and Android platforms. The primary interface, as displayed in Figure 3, encompasses functionalities such as disinfection settings and control, mobile robot control, status display, and map management. The operators are able to manipulate the disinfection robot in a secure environment to accomplish disinfection tasks. The system allows for the importing of Drawing Exchange Format (DXF) map files (ASCII or binary format files of CAD graphics files) to use them as maps, reducing the disinfection work preparation time significantly. This feature greatly improves the work efficiency of the autonomous mobile disinfection robot. An emergency protection feature was developed to guarantee safety by allowing the robot to return to a pre-set safe point automatically when it loses contact. The mobile robot is based on the Robot Operating System (ROS) and incorporates Simultaneous Localization and Mapping (SLAM) technology to construct a map of the environment. Employing global and local path planning, as well as robot motion control, the disinfection robot autonomously navigates to designated disinfection points while avoiding obstacles.

### 2.3. Disinfection Modes

There are two available disinfection modes: continuous disinfection and fixed-point disinfection. In continuous disinfection mode, the robot moves continuously and conducts disinfection based on the modeling results until the disinfection task is completed. The fixed-point disinfection mode requires several disinfection points to be set in advance. The disinfection robot then navigates to each point in sequence, automatically avoiding obstacles along the way. Finally, each point is disinfected in four directions—front, back, left, and right—based on the results of the fixed-point disinfection modeling.

## 3. Experimental Design

### 3.1. Experimental Setup

#### 3.1.1. Experimental Materials and Configurations

The experimental materials consist of the autonomous mobile disinfection robot, a ZR-2000 intelligent air microbial sampler, an electric heating constant-temperature biochemical incubator, as well as tryptone soybean agar (TSA), 1% sodium thiosulfate neutralizer, and 1% hydrogen peroxide solution.

The disinfection robot has the following dimensions: 0.52 m in length, 0.52 m in width, and 1.44 m in height. It is powered by a 24-volt battery. The maximum load capacity of the wheels is 60 kg, with a maximum obstacle clearance height of 3 cm. The robot can traverse slopes with a maximum inclination of 10 degrees and achieve a maximum speed of 0.7 m/s. It is equipped with four UV lamps, each with a length of 89.5 cm and a wavelength of 253.7 nm, providing a radiation intensity of 135 μW/cm^2^ at a distance of 1 m. There are four ultrasonic atomizers, each with a misting rate of 500 mL/h. The liquid tank has a capacity of approximately 8 L.

The industrial computer is equipped with an Intel(R) Celeron(R) CPU N3160 @1.60 GHz, operating on Ubuntu 16.04 and ROS Kinetic systems. The laser radar is the RPlidar S2 from Shanghai Slamtec Co., Ltd. (Shanghai, China). The IMU is the CH104M from Beijing Hipnuc Electronic Technology Co., Ltd. (Beijing, China).

#### 3.1.2. Experiment Environment

**Figure 4:**
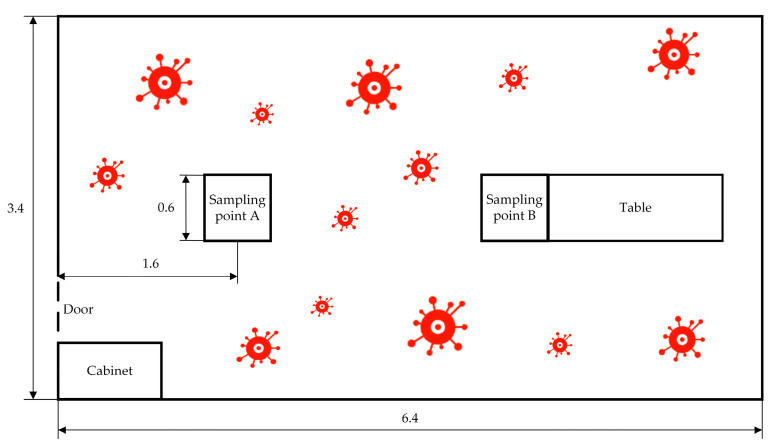
Schematic diagram of the experimental site layout (unit/meter).

The test was conducted in an office measuring 6.4 m in length, 3.4 m in width, and 2.8 m in height, as illustrated in Figure 4. Following the guidelines outlined in the “Technical Standard for Disinfection” (2002 edition) [13], two sampling points (A and B) were selected for testing, with the sampler placed at a height of 1 m above the ground. The temperature was 20–23 °C, and the relative humidity was kept within the range of 50–60%.

#### 3.1.3. Effective Disinfectant Dosage

The effectiveness of UV disinfection primarily relies on the dosage of UV radiation. According to the Bunsen–Roscoe law [14], disinfection dosage refers to the accumulation of radiation intensity over time:

(1) $$K=I_{d}\cdot t$$

In the formula, *K* represents the UV disinfection dosage; $I_{d}$ represents the radiation intensity at *d* meters away from the UV lamps; and *t* represents the disinfection duration. Table 1 shows the UV dosages required to eliminate various common germs.

A UV dose of 3700 μJ/m^2^ can inactivate 99.9% of the SARS-CoV-2 virus, while a dose of 16,900 μJ/m^2^ can achieve near-complete inactivation [16]. Within the effective disinfection range and duration of the disinfection robot, the UV radiation dose *K* is 20,000 μJ/m^2^.

#### 3.1.4. Disinfection Rate Calculation

According to the “Technical Standard for Disinfection” (2002 edition), the calculation formula for the average number of natural bacteria in the air before and after disinfection is as follows:

(2) $$\mathrm{Air}\;\mathrm{bacteria}\;\mathrm{content}({\mathrm{cfu}/m}^{3})=\frac{\mathrm{The}\;\mathrm{total}\;\mathrm{number}\;\mathrm{of}\;\mathrm{bacteria}\;\mathrm{on}\;\mathrm{the}\;\mathrm{six-}\mathrm{level}\;\mathrm{sampling}\;\mathrm{plate}(\mathrm{cfu})}{\mathrm{Sampling}\;\mathrm{rate}(L/\min)\times \mathrm{Sampling}\;\mathrm{duration}(\min)}\times 1000$$

The disinfection rate of natural bacteria in the air is as follows:

(3) $$\begin{array}{l} \mathrm{Disinfection}\;\mathrm{rate}\;(\%)=\\ \frac{\mathrm{Mean}\;\mathrm{bacterial}\;\mathrm{count}\;\mathrm{before}\;\mathrm{disinfection}-\mathrm{Mean}\;\mathrm{bacterial}\;\mathrm{count}\;\mathrm{after}\;\mathrm{disinfection}}{\mathrm{Mean}\;\mathrm{bacterial}\;\mathrm{count}\;\mathrm{before}\;\mathrm{disinfection}}\times 100\% \end{array}$$

### 3.2. Disinfection Model

Due to the significant role played by UV lamps in disinfection, a mathematical model was established for UV radiation disinfection.

#### 3.2.1. The Continuous Disinfection Model

In the continuous disinfection mode, the effectiveness of disinfection mainly relies on the moving speed and the robot path. The radiation intensity of any point in the space radiation field created by multiple UV lamps follows the principle of superposition [17]. Therefore, the radiation intensity $I_{1}$ of a group of UV lamps with four lamps tubes placed side by side at a distance of 1 m is four times that of a single UV lamp. Moreover, the intensity of UV radiation satisfies the “inverse square law” [18,19], meaning that the radiation intensity $I_{d}$ is inversely proportional to the square of the distance *d*:

(4) $$I_{d}=\frac{I_{1}}{d^{2}}$$

When the radiation intensity is below 70 μW/cm^2^, the disinfection efficacy remains suboptimal, even with prolonged exposure [20]. Therefore, the maximum effective disinfection distance $L_{\max}$ of the UV lamp assembly on the disinfection robot is 2.8 m.

(1) Disinfecting the area straight ahead

**Figure 5:**
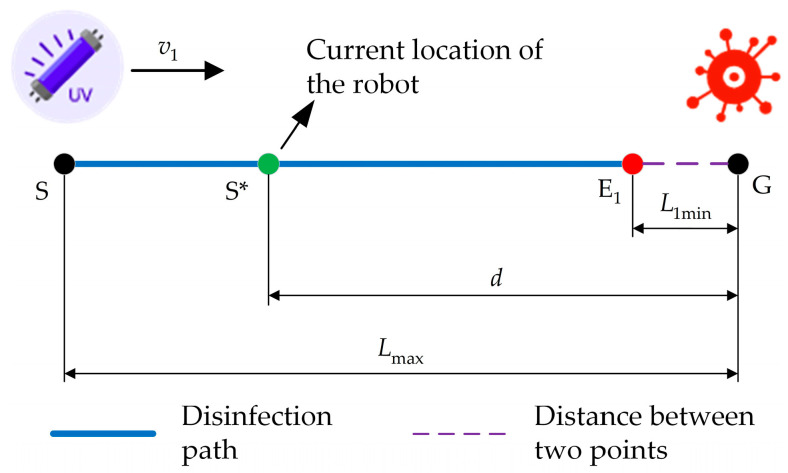
Schematic diagram of the process of disinfecting the area directly in front of the robot.

When disinfecting the area in front of the robot, the shortest effective disinfection distance $L_{1\min}$ is the distance between the UV lamp group and the front contour of the robot, which is 0.1 m. The disinfection process is illustrated in Figure 5. The disinfection robot starts from point S and disinfects point G in front of it at a constant speed $v_{1}$. The cumulative disinfection dose $K_{1}$ of the disinfection robot on point G during its movement from the farthest disinfection point S to the nearest disinfection point $E_{1}$ is as follows:

(5) $$K_{1}=\int_{0}^{t_{1\mathrm{total}}}\frac{I_{1}}{\left(L_{\max}-v_{1}t\right)}dt$$

In the formula, $t_{1\mathrm{total}}$ represents the total duration for the robot to move from the farthest disinfection point S to the nearest disinfection point $E_{1}$. It can be concluded that the robot’s maximum speed is 0.26 m/s.

(2) Disinfecting the area at the front side

**Figure 6:**
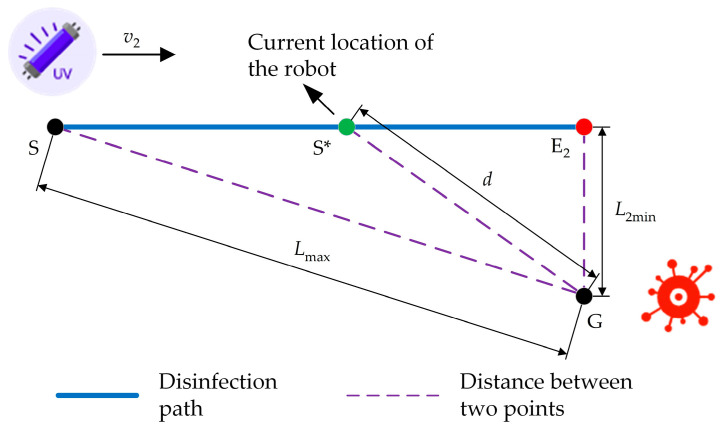
Schematic diagram of disinfecting the area at the front side of the robot.

When disinfecting the area at the front side of the robot, the shortest effective disinfection distance $L_{2\min}$ is the distance between the UV lamp group and the side contour of the robot, which is 0.3 m. As shown in Figure 6, during the process of the disinfection robot moving from the farthest disinfection point S to the nearest disinfection point $E_{2}$ at a constant speed $v_{2}$, the accumulated disinfection dose $K_{2}$ on point G is as follows:

(6) $$K_{2}=\int_{0}^{t_{2\mathrm{total}}}\frac{I_{1}}{{\left(\sqrt{L_{\max}{}^{2}-L_{2\min}{}^{2}}-v_{2}t\right)}^{2}+L_{2\min}{}^{2}}dt$$

In the formula, $t_{2\mathrm{total}}$ is the total duration for the robot to move from the farthest disinfection point S to the nearest disinfection point $E_{2}$. It can be obtained that the maximum moving speed of the robot is 0.13 m/s when disinfecting the area at its front side. As a result, in order to achieve optimal disinfection results when using the continuous disinfection mode, the robot’s moving speed should not exceed 0.13 m/s.

(3) Disinfection path

**Figure 7:**
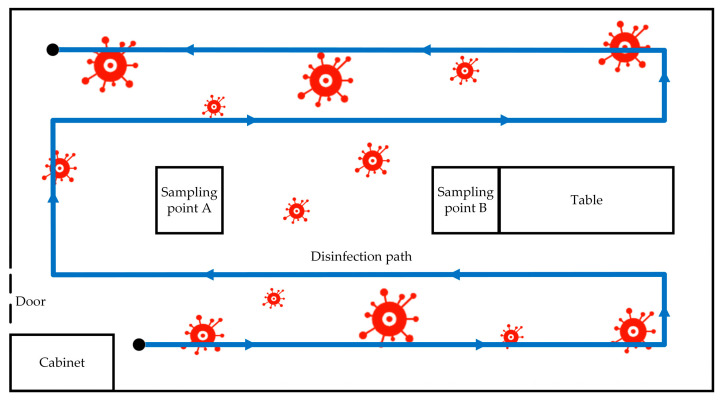
Schematic diagram of continuous disinfection.

Taking into account the impact of the width of the disinfection robot and its turning radius, when the robot disinfects along the wall, it is set to be 0.4 m away from the wall, and the path interval is 0.6 m. The disinfection path of the robot under the continuous disinfection mode can be obtained as shown in Figure 7.

#### 3.2.2. The Fixed-Point Disinfection Model

**Figure 8:**
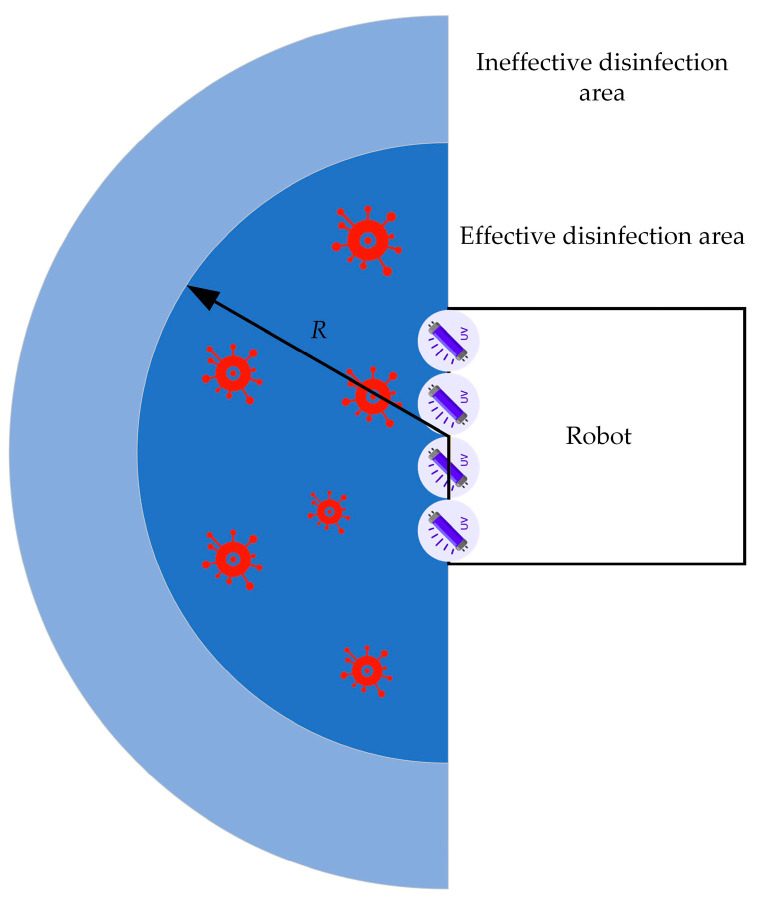
The effective disinfection area of the UV lamp group.

The effectiveness of fixed-point disinfection relies heavily on the number of disinfection points and the duration of disinfection at each point. Figure 8 illustrates the effective disinfection area of the UV lamp group in the disinfection robot. Any position within this area can receive a UV radiation dose of up to 20,000 μJ/m^2^. As the robot needs to disinfect in four directions—front, back, left, and right—upon reaching each disinfection point, we can approximate the total area *S* of the effective disinfection area of each point using the following formula:

(7) $$S=\pi {\left(R+\frac{r_{w}}{2}\right)}^{2}-r_{w}{}^{2}$$

In the formula, *R* represents the radius of the effective disinfection area of the UV lamp group; $r_{w}$ represents the width of the edge where the robot UV lamp group is located.

The room has an area of 21.76 square meters, and 10 disinfection points have been selected. To achieve the complete coverage of the room, *R* has been calculated as 0.63 m. By combining Equations (1) and (4), the total disinfection duration $t_{\mathrm{total}}$ for each point can be determined as follows:

(8) $$t_{\mathrm{total}}=\frac{K\cdot R^{2}}{I_{1}}$$

**Figure 9:**
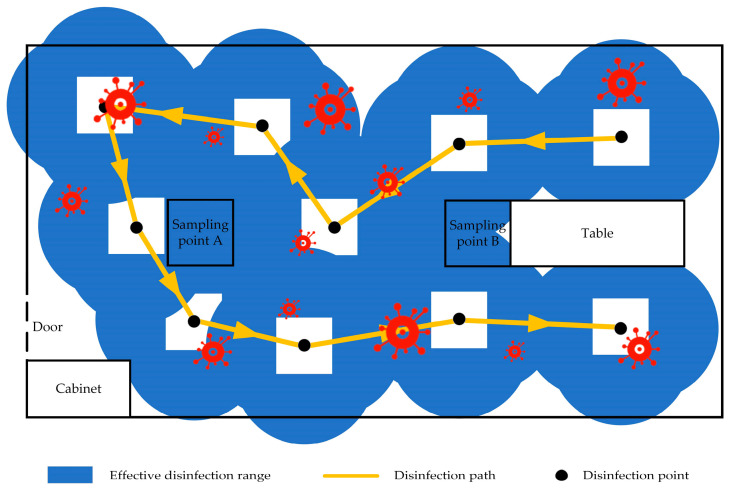
Schematic diagram of fixed-point disinfection.

The solution duration for the given problem is 15 s. It is important to note that the model presented above is only applicable to the space at the same height as the UV lamp group. Additionally, the length of the UV lamp is only one third of the height of the room. As a result, the total disinfection duration for each point is determined as 48 s, distributing evenly across all directions (front, back, left, and right), with 12 s of disinfection duration allocated to each direction. The disinfection path of the autonomous mobile disinfection robot in the fixed-point disinfection mode is illustrated in Figure 9.

## 4. Experiment Results

After the disinfection was completed, the collected samples were cultured in a constant temperature incubator at 37 °C for 48 h, followed by bacterial identification and colony counting. The test results are shown in Table 2.

## 5. Analysis and Discussion

Table 3 presents a summary of on-site disinfection efficacies derived from using three different methods: UV radiation disinfection, hydrogen peroxide disinfection, and a combination of UV lamp and hydrogen peroxide disinfection. To disinfect 90.58% natural bacteria in the air, a concentration of 0.18 mL/m^3^ H_2_O_2_ must be present in the room for at least 30 min [21]. This duration only accounts for the action duration of the disinfectant and not its spray duration. In our combination disinfection robot, the H_2_O_2_ disinfectant is sprayed at a total speed of 2000 mL/h, taking approximately 33 min to reach 0.18 mL/m^3^ concentration. Considering the disinfectant’s action duration, to achieve a disinfection rate of over 90% for airborne natural bacteria, if relying solely on hydrogen peroxide aerosol disinfection, the actual required spray and total duration of action would significantly exceed the initial 33 min. Furthermore, UV lamps with a total radiation intensity of 710 μW/cm^2^ require at least 40 min to achieve 92.57% disinfection rate [22]. The total radiation intensity of the UV lamp assembly in the disinfection robot designed in this study is 540 μW/cm^2^, achieving a 92.95% eradication rate of airborne natural bacteria in just 30 min. Not only does it surpass the national health standard of eliminating 90% natural bacteria in the air within 2 h [23] but also the disinfection efficiency is much higher than that of traditional UV lamp air disinfection and hydrogen peroxide spray disinfection.

The length of the UV lamps is approximately one third of the room height, indicating that approximately two-thirds of the air in the room is not effectively exposed to UV radiation. Furthermore, in a sealed room, the air flow rate is low and the exchange efficiency is poor. These factors collectively contribute to a reduction in the eradication rate of airborne natural bacteria. It is worth noting that the target of disinfection in this study is the entire airborne natural bacteria in the environment. Unlike the direct exposure of objects or microbial agents to UV lamps at close range, the disinfection efficiency between the two scenarios is not comparable.

By comparing the UV radiation doses required to eliminate various common germs, it was determined that, in order to achieve a disinfection rate of 99.99% for airborne natural bacteria, the UV radiation dose should not be less than 20,000 μJ/m^2^. On this basis, the effective disinfection range of the robot was calculated to be a semi-circular area with a radius of 2.8 m in front of the UV lamp assembly. Taking aerosol disinfection into account, the actual effective disinfection range will be even larger and not limited solely to the frontal area of the robot.

A quantitative analysis revealed that the movement speed of the disinfection robot in continuous disinfection mode should not exceed 0.13 m/s, and in the fixed-point disinfection model, the disinfection duration for each direction at each point should not be less than 12 s. While these parameters may not be universally applicable to all disinfection environments, the modeling and analytical approaches presented herein offer a conceptual framework for researchers in this field.

In practical application, the selection of disinfection strategies should be flexible, considering the specific environmental conditions and requirements. In open spaces, priority may be given to the faster continuous disinfection mode. Conversely, in complex environments with numerous obstacles, the fixed-point disinfection model should be chosen. In environments with a high pathogen load, such as hospitals or outbreak areas, where the comprehensive eradication of airborne natural bacteria is imperative, adjustments can be made by reducing the intervals of disinfection paths in continuous disinfection mode or by increasing the number of disinfection points in the fixed-point disinfection model. Additionally, extending the disinfection duration may also be considered to meet the required disinfection standards.

## 6. Conclusions

Traditional UV radiation disinfection tends to have dead corners, leading to incomplete disinfection. In contrast, low-concentration hydrogen peroxide aerosol disinfection offers comprehensive and environmentally friendly advantages. To address this, we propose an autonomous mobile combination disinfection system wherein UV radiation disinfection serves as the primary method and low-concentration hydrogen peroxide aerosol disinfection serves as a supplementary method. Through a comprehensive analysis and designing both hardware and software components, we have successfully developed a prototype for an autonomous mobile combination disinfection robot. By establishing models of UV radiation disinfection processes in the continuous and fixed-point modes, we quantitatively computed the effective disinfection range, speed, and duration of the disinfection robot. The prototype underwent on-site testing by a professional third-party testing agency in an office measuring 22 square meters with a height of 2.8 m. The results demonstrate that the combined disinfection system achieved a 92.95% eradication rate of airborne natural bacteria within just 30 min. In comparison to standalone UV radiation disinfection, the efficiency is increased by at least 25% and is significantly superior to standalone hydrogen peroxide aerosol disinfection. The autonomous mobile combination disinfection system enables the green, rapid, efficient, and all-encompassing disinfection of airborne natural bacteria.

## Figures and Tables

**Table 1:** Approximate UV dosages required for eliminating various common germs [15].

Germs	Dosages Required for Different Disinfection Rates (μJ/m^2^)		
	90%	99%	99.99%
Influenza virus	1000	2000	>5000
Escherichia coli	3000	6000	12,000
Shigella dysenteriae	3000	6000	12,000
Staphylococcus aureus	3000	6000	12,000
Salmonella typhi	4000	8000	16,000
Mycobacterium tuberculosis	5000	10,000	20,000
Corynebacterium diphtheriae	5000	10,000	20,000

**Table 2:** Disinfection test results.

Test Number	Disinfection Method	Disinfection Duration	Sampling Point	Number of Bacteria before Disinfection (cfu/m^3^)	Number of Bacteria after Disinfection (cfu/m^3^)	Disinfection Rate (%)	Average Disinfection Rate (%)
1	Continuous disinfection	5 min	Point A	417	141	66.19	71.83
			Point B	304	92	69.74	
2	Continuous disinfection	5 min	Point A	219	78	64.38	
			Point B	269	35	86.99	
3	Fixed-point disinfection	15 min	Point A	544	127	76.65	81.21
			Point B	410	78	80.98	
4	Fixed-point disinfection	15 min	Point A	375	49	86.93	
			Point B	608	120	80.26	
5	Fixed-point disinfection	30 min	Point A	375	27	**92.80**	**92.95**
			Point B	608	42	**93.09**	

**Table 3:** The on-site disinfection efficacies of different methods.

Disinfection Method	Disinfection Duration (min)	Disinfection Rate (%)	Explanation	Reference
Hydrogen peroxide	At least 30	90.58	The concentration of H_2_O_2_ in the room is 0.18 mL/m^3^	21
UV	40	92.57	The total radiation intensity of the UV lamps is 710 μW/cm^2^	22
Combined fixed-point disinfection	30	**92.95**	The total radiation intensity of the UV lamps is 540 μW/cm^2^	Ours

## Data Availability

No new data were created or analyzed in this study. Data sharing is not applicable to this article.
